# Simulating Solid-Liquid Phase-Change Heat Transfer in Metal Foams via a Cascaded Lattice Boltzmann Model

**DOI:** 10.3390/e24030307

**Published:** 2022-02-22

**Authors:** Xiang-Bo Feng, Qing Liu

**Affiliations:** 1 Xi’an Key Laboratory of Advanced Photo-Electronics Materials and Energy Conversion Device, School of Science, Xijing University, Xi’an 710123, China; fengxiangbo@xjtu.edu.cn; 2 School of Energy and Power Engineering, Xi’an Jiaotong University, Xi’an 710049, China; 3 School of Resources Engineering, Xi’an University of Architecture and Technology, Xi’an 710055, China

**Keywords:** cascaded collision model, lattice Boltzmann model, melting, enthalpy methodology, metal foams

## Abstract

A cascaded lattice Boltzmann (CLB) model is constructed for simulating heat transfer in metal-foam-based solid-liquid phase change materials (PCMs). The present model captures the phase interface implicitly via the enthalpy methodology, and to avoid iterations in simulations, the CLB equation of the PCM employs the enthalpy as the basic evolution variable through modifying the cascaded collision process. Numerical results demonstrate the effectiveness and practicability of the CLB model for investigating heat transfer in solid-liquid PCMs with metal foams. The effects of the inertial coefficient, permeability and porosity on the melting process are investigated. The results indicate that the empirical correlations of inertial coefficient and permeability based on packed beds overestimate the melting rate at high porosities. Moreover, the porosity has significant impact on phase-change processes. The melting rate increases as the porosity of the metal foam decreases since heat conduction through high thermal conductive metal foam dominates the total heat transfer.

## 1. Introduction

Latent heat storage (LHS) using solid-liquid PCMs attracts wide attention from the academic research community and industry field due to its importance for solving energy and environment issues [1,2,3,4]. Although solid-liquid PCMs have some outstanding advantages (i.e., high energy storage density), they usually suffer from low thermal conductivities (in the range of 0.1~0.6 W/(m·K) [5]), which strongly affects the LHS system’s thermal efficiency. To enhance PCMs’ thermal conductivities, embedding high thermal conductive metal foams within PCMs to form composite PCMs has long been practiced [6,7]. In the past several decades, various conventional numerical methods (e.g., FVM [8,9]) have been developed to investigate thermal behaviors of heat transfer in solid-liquid PCMs with metal foams. The lattice Boltzmann (LB) method [10], as a particle-based numerical tool evolved from lattice-gas automata [11], has also attracted great attention in studying heat transfer in solid-liquid PCMs [12].

The LB method is a mesoscopic modeling method sitting in the intermediate zone between macroscopic continuum-based methods and microscopic molecular dynamics method. This scale-bridging nature is a fundamental feature of the LB method, and consequently, it has several distinctive advantages, such as local nature of operations, ideal for parallel computing, avoiding solving Poisson equation for incompressible flows, and easy implementation of complex boundary conditions with elementary mechanical rules [13,14]. Due to its inherent advantage, the LB method is particularly effective in studying transient phase-change processes with complex interfacial dynamics and moving phase interface. Moreover, for transient phase-change processes, the efficiency of the LB method is higher than that of the conventional numerical methods if time steps are equal. In 1998, Fabritiis et al. [15] developed the first LB scheme for investigating solid-liquid phase change. Since then, many LB models have been constructed for transient solid-liquid phase-change problems, and these models can be divided into three major groups: phase-field approach [16,17], enthalpy-based approach [18,19,20], and interface-tracking approach [21,22]. Among the three types of phase-change LB models, the enthalpy-based approach has attracted great attention owing to its effectiveness and easy implementation in modeling solid-liquid phase-change processes. In the enthalpy-based approach, the diffusive phase interface as well as the liquid and solid phases are distinguished via liquid fraction, which makes this approach simple and effective. In the past decade, the enthalpy-based approach has also been successfully adopted to investigate solid-liquid phase change in porous media by many researchers [23,24,25,26,27,28]. These enthalpy-based LB models are mainly constructed based on Bhatnagar–Gross–Krook (BGK) [23,24,25] and multiple-relaxation-time (MRT) [26,27,28] models. As reported in Ref. [27], the effect of unphysical numerical diffusion on numerical simulations can be serious in the BGK model, while the MRT model can effectively eliminate such effect since it has sufficient tunable relaxation parameters.

In addition to BGK and MRT collision models, the cascaded collision model [29,30,31,32] also attracts significant attention in the LB community. The cascaded collision model was first developed by Geier et al. [29] in the year of 2006. In the framework of this model, the collision step is executed based on central moments, and different central moments are relaxed to their equilibria with different relaxation rates. In the CLB method, a high degree of Galilean invariance can be preserved since the influence between different orders of moments has been eliminated [29,30]. For heat transfer in solid-liquid PCMs with metal foams, the interfacial behaviors in phase-change region are very important, and interfacial heat transfer (thermal non-equilibrium effect) plays a significant role because metal foam’s thermal conductivity is usually three orders of magnitude higher than PCM’s thermal conductivity. An in-depth understanding of the complex interfacial dynamics and thermal behaviors requires efficient and powerful numerical tool. Since the cascaded collision model has tunable relaxation parameters as well as a high degree of Galilean invariance, it is expected that the complex interfacial dynamics and thermal behaviors of heat transfer in metal-foam-based solid-liquid PCMs can be well captured by using the cascaded collision model. Hence, this work aims to propose a CLB model for simulating heat transfer in solid-liquid PCMs with metal foams, in which the enthalpy methodology is adopted to capture the phase interface implicitly. Moreover, the effects of the inertial coefficient, permeability and porosity on melting process will be investigated.

## 2. Macroscopic Governing Equations

For heat transfer in metal-foam-based solid-liquid PCMs, the macroscopic governing equations are given by [9,12,23]:

(1) ∇·u=0

(2) $$\frac{\partial u}{\partial t}+\left(u\cdot \nabla \right)\left(\frac{u}{\phi }\right)=-\frac{1}{\rho_{f}}\nabla \left(\phi p\right)+v_{e}\nabla^{2}u+F$$

(3) $$\frac{\partial }{\partial t}\left(\phi \rho_{f}c_{pf}T_{f}\right)+\nabla \cdot \left(\rho_{l}c_{pl}T_{f}u\right)=\nabla \cdot \left[\left(k_{ef}+k_{d}\right)\nabla T_{f}\right]+h_{mf}a_{mf}\left(T_{m}-T_{f}\right)-\underline{\frac{\partial }{\partial t}\left(\phi \rho_{l}L_{a}f_{l}\right)}$$

(4) $$\frac{\partial }{\partial t}\left[\left(1-\phi \right)\rho_{m}c_{pm}T_{m}\right]=\nabla \cdot \left(k_{em}\nabla T_{m}\right)+h_{mf}a_{mf}\left(T_{f}-T_{m}\right)$$

Equations (1) and (2) are the so-called generalized non-Darcy equations. The last term of Equation (3) is the phase-change term ($f_{l}=0$ denotes the solid region, $0<f_{l}<1$ denotes the phase-change region, and $f_{l}=1$ denotes the liquid region).

The total body force F is determined by:

(5) $$F=-\frac{\phi v_{fl}}{K}u-\frac{\phi F_{\phi }}{\sqrt{K}}\left|u\right|u+\phi G$$

According to the Boussinesq approximation, the body force G is given by $G=-g\beta \left(T-T_{0}\right)$.

For flow through packed bed consisting of spherical particles, $F_{\phi }$ and K are given by:

(6) $$F_{\phi }=\frac{1.75}{\sqrt{150\phi^{3}}},\;K=\frac{\phi^{3}d_{p}^{2}}{150{\left(1-\phi \right)}^{2}}$$

$d_{p}^{}$ in Equation (6) denotes the mean particle diameter.

For metal foams such as aluminum foam, $F_{\phi }$ and K can be well predicted by the following empirical correlations [33]:

(7) $$F_{\phi }=A_{1}{\left(1-\phi \right)}^{b_{1}}{\left(\frac{d_{f}}{d_{p}}\right)}^{c_{1}},\;K=A_{2}{\left(1-\phi \right)}^{b_{2}}d_{p}^{2}{\left(\frac{d_{f}}{d_{p}}\right)}^{c_{2}}$$

where $A_{1}=0.00212$, $b_{1}=-0.132$, $c_{1}=-1.63$, $A_{2}=0.00073$, $b_{2}=-0.224$, and $c_{2}=-1.11$. $d_{p}^{}$ in Equation (7) denotes the mean diameter of the pores. 

An appropriate equation for the structure of the metal foam is given by [33]:

(8) $$\frac{d_{f}}{d_{p}}=1.18\sqrt{\frac{1-\phi }{3\pi }}\frac{1}{1-e^{-\left(1-\phi \right)/0.04}}$$

The effective thermal conductivities $k_{ef}$ and $k_{em}$ can be determined by analytical models [33,34]. Since the metal foam’s thermal conductivity is rather high, the thermal dispersion can be neglected and $k_{d}$ is usually set to in Equation (3) [8].

$a_{mf}$ can be predicted by the following relation [35]:

(9) $$a_{mf}=\frac{3\pi d_{f}}{{\left(0.59d_{p}\right)}^{2}}\left[1-e^{-\left(1-\phi \right)/0.04}\right]$$

$h_{mf}$ depends on the foam structure, Reynolds number, and Prandtl number. For forced convective flow in metal foams, several empirical correlations for $h_{mf}$ have been proposed [8,34]. For natural convection flow through metal foams, the following empirical correlation is widely used [35]:

(10) $$h_{mf}=\frac{k_{f}}{d_{f}}\left(0.36+\frac{0.518Ra_{d}^{1/4}}{{\left[1+{\left(0.559/Pr\right)}^{9/16}\right]}^{4/9}}\right)$$

where $Ra_{d}=g\beta \Delta Td_{f}^{3}/(\alpha_{fl}v_{fl})$, in which $\alpha_{fl}=k_{fl}/\left(\rho_{l}c_{pl}\right)$.

## 3. Enthalpy-Based CLB Model for Solid-Liquid Phase Change in Metal Foams

For the flow field described by Equations (1) and (2), the isothermal CLB equation in Ref. [32] is adopted. In what follows, the enthalpy-based CLB equation for PCM’s temperature field and the thermal MRT-LB equation for metal foam’s temperature field are presented in detail. The D2Q5 lattice is adopted, in which the discrete velocities $\left\{e_{i}|i=0,\;\ldots ,\;4\right\}$ are given by:

(11) $$e_{i}=\{\begin{array}{ll} \left(0,0\right), & i=0\\ \left(\cos\left[\left(i-1\right)\pi /2\right],\sin\left[\left(i-1\right)\pi /2\right]\right)c, & i=1\sim 4 \end{array}$$

### 3.1. Enthalpy-Based CLB Equation

#### 3.1.1. Thermal CLB Equation for Fluid Phase without Phase-Change Term

Without the phase-change term, Equation (3) can be rewritten as:

(12) $$\frac{\partial }{\partial t}\left(c_{pf}T_{f}\right)+\nabla \cdot \left(\frac{c_{pf}T_{f}u}{\phi }\right)=\nabla \cdot \left(\frac{k_{ef}}{\phi \rho_{f}}\nabla T_{f}\right)+\frac{h_{mf}a_{mf}\left(T_{m}-T_{f}\right)}{\phi \rho_{f}}$$

We first introduce the thermal CLB equation for solving Equation (3) without phase-change term based on the simplified CLB method [30]. The raw moments $\left\{k_{mn}^{T}\right\}$ and central moments $\left\{{\tilde{k}}_{mn}^{T}\right\}$ of the discrete temperature distribution function $g_{fi}$ of the fluid phase are defined as follows [30]:

(13) $$k_{mn}^{T}=\langle g_{fi}|e_{ix}^{m}e_{iy}^{n}\rangle ,\;{\tilde{k}}_{mn}^{T}=\langle g_{fi}|{\left(e_{ix}^{}-u_{x}\right)}^{m}{\left(e_{iy}^{}-u_{y}\right)}^{n}\rangle$$

The simplified raw-moment $|n_{fi}^{}\rangle$ and central-moment $|{\tilde{n}}_{fi}^{}\rangle$ are given by [30]

(14) $$|n_{fi}^{}\rangle ={\left[k_{00}^{T},k_{10}^{T},k_{01}^{T},k_{20}^{T},k_{02}^{T}\right]}^{{}^{T}},\;|{\tilde{n}}_{fi}^{}\rangle ={\left[{\tilde{k}}_{00}^{T},{\tilde{k}}_{10}^{T},{\tilde{k}}_{01}^{T},{\tilde{k}}_{20}^{T},{\tilde{k}}_{02}^{T}\right]}^{{}^{T}}$$

Through the transformation matrix $M_{T}$ and shift matrix $N_{T}$, $\left\{n_{fi}^{}\right\}$ and $\left\{{\tilde{n}}_{fi}^{}\right\}$ can be determined by [30]

(15) $$|{\tilde{n}}_{fi}^{}\rangle =N_{T}|n_{fi}^{}\rangle ,\;|n_{fi}^{}\rangle =M_{T}|g_{fi}^{}\rangle$$

The thermal CLB equation for solving Equation (12) can be divided into two parts: collision and streaming processes. Through shifting procedure ($|{\tilde{n}}_{fi}^{}\rangle =N_{T}|n_{fi}^{}\rangle$), collision process is implemented in central-moment space as

(16) $$|{\tilde{n}}_{fi}^{*}\rangle =|{\tilde{n}}_{fi}^{}\rangle -\Theta \left(|{\tilde{n}}_{fi}^{}\rangle -|{\tilde{n}}_{fi}^{eq}\rangle \right)+|{\tilde{S}}_{fi}^{}\rangle$$

where $\left\{{\tilde{n}}_{fi}^{eq}\right\}$ are equilibrium central moments, $\left\{{\tilde{n}}_{fi}^{*}\right\}$ denotes the post-collision central moments, $\Theta =\mathrm{diag}\left(\zeta_{T},\zeta_{\alpha },\zeta_{\alpha },\zeta_{e},\zeta_{e}\right)$ is the relaxation matrix, $|{\tilde{S}}_{fi}^{}\rangle$ is the source term.

The streaming process is still implemented in velocity space as

(17) $$g_{fi}\left(x+e_{i}\delta_{t},\;t+\delta_{t}\right)=g_{fi}^{*}\left(x,\;t\right)$$

where $g_{fi}^{*}$ is determined by $|g_{fi}^{*}\rangle =M_{T}^{-1}N_{T}^{-1}|{\tilde{n}}_{fi}^{*}\rangle$ ($|g_{fi}^{*}\rangle =M_{T}^{-1}|n_{fi}^{*}\rangle$ and $|n_{fi}^{*}\rangle =N_{T}^{-1}|{\tilde{n}}_{fi}^{*}\rangle$).

$M_{T}$ and $N_{T}$ are given by (c=1):

(18) $$M_{T}=\left[\begin{array}{rrrrr} 1 & 1 & 1 & 1 & 1\\ 0 & 1 & 0 & -1 & 0\\ 0 & 0 & 1 & 0 & -1\\ 0 & 1 & 0 & 1 & 0\\ 0 & 0 & 1 & 0 & 1 \end{array}\right]$$

(19) $$N_{T}=\left[\begin{array}{rrrrr} 1 & 0 & 0 & 0 & 0\\ -u_{x}/\phi & 1 & 0 & 0 & 0\\ -u_{y}/\phi & 0 & 1 & 0 & 0\\ u_{x}^{2} & -2\phi u_{x} & 0 & 1 & 0\\ u_{y}^{2} & 0 & -2\phi u_{y} & 0 & 1 \end{array}\right]$$

Explicitly, the equilibrium central moments $\left\{{\tilde{n}}_{fi}^{eq}\right\}$ and raw moments $\left\{n_{fi}^{eq}\right\}$ are given by:

(20) $$|{\tilde{n}}_{fi}^{eq}\rangle ={\left[c_{pf}T_{f},0,0,c_{sf}^{2}c_{pf}T_{f},c_{sf}^{2}c_{pf}T_{f}\right]}^{T}$$

(21) $$|n_{fi}^{eq}\rangle ={\left[c_{pf}T_{f},\frac{c_{pf}T_{f}u_{x}}{\phi },\frac{c_{pf}T_{f}u_{y}}{\phi },c_{sf}^{2}c_{pf}T_{f}+u_{x}^{2}c_{pf}T_{f},c_{sf}^{2}c_{pf}T_{f}+u_{y}^{2}c_{pf}T_{f}\right]}^{T}$$

The equilibrium temperature distribution function $g_{fi}^{eq}$ can be determined by $|g_{fi}^{eq}\rangle =M_{T}^{-1}|n_{fi}^{eq}\rangle$.

Explicitly, the source term $|{\tilde{S}}_{fi}^{}\rangle$ is given by:

(22) $$|{\tilde{S}}_{fi}^{}\rangle ={\left[Sr_{f}+\frac{1}{2}\delta_{t}\partial_{t}Sr_{f},0,0,0,0\right]}^{T}$$

where $Sr_{f}=h_{mf}a_{mf}\left(T_{m}-T_{f}\right)/\left(\phi \rho_{f}\right)$.

The temperature $T_{f}$ is defined as:

(23) $$c_{pf}T_{f}=\sum_{i=0}^{4}g_{fi}$$

The effective thermal diffusivity $\alpha_{ef}$ is:

(24) $$\alpha_{ef}=\frac{k_{ef}}{\phi \rho_{f}c_{pf}}=c_{sf}^{2}\left(\zeta_{\alpha }^{-1}-\frac{1}{2}\right)\delta_{t}$$

#### 3.1.2. Enthalpy-Based CLB Equation for PCM with Phase Change

The enthalpy-based CLB equation for solving Equation (3) is developed based on the thermal CLB equation in Section 3.1.1. By combining the phase-change term $\partial_{t}\left(\phi \rho_{l}L_{a}f_{l}\right)$ with the transient term $\partial_{t}\left(\phi \rho_{f}c_{pf}T_{f}\right)$ in Equation (3), we can obtain:

(25) $$\frac{\partial H_{f}}{\partial t}+\nabla \cdot \left(\frac{c_{pl}T_{f}u}{\phi }\right)=\nabla \cdot \left(\frac{k_{ef}}{\phi \rho_{l}}\nabla T_{f}\right)+\frac{h_{mf}a_{mf}\left(T_{m}-T_{f}\right)}{\phi \rho_{l}}$$

where $H_{f}=\sigma c_{pl}T_{f}+L_{a}f_{l}$ is the enthalpy, and $\sigma =\frac{\rho_{f}c_{pf}}{\rho_{l}c_{pl}}=\frac{f_{l}\rho_{l}c_{pl}+\left(1-f_{l}\right)\rho_{s}c_{ps}}{\rho_{l}c_{pl}}$ is the heat capacity ratio. In the liquid phase ($f_{l}=1$), $H_{f}=c_{pl}T_{f}+L_{a}$ and $\sigma_{l}=1$; in the solid phase ($f_{l}=0$), $H_{f}=\sigma_{s}c_{pl}T_{f}$ and $\sigma_{s}=\frac{\rho_{s}c_{ps}}{\rho_{l}c_{pl}}$. The source term $Sr_{f}=h_{mf}a_{mf}\left(T_{m}-T_{f}\right)/\left(\phi \rho_{l}\right)$.

Formally, the collision and streaming processes of the CLB equation for solving Equation (25) are still given by Equations (16) and (17), respectively. However, to match the enthalpy-based energy Equation (25), the equilibrium central moments $\left\{{\tilde{n}}_{fi}^{eq}\right\}$ and raw moments $\left\{n_{fi}^{eq}\right\}$ should be chosen as follows:

(26) $$|{\tilde{n}}_{fi}^{eq}\rangle ={\left[H_{f},0,0,c_{sf}^{2}c_{pl}T_{f},c_{sf}^{2}c_{pl}T_{f}\right]}^{T}$$

(27) $$|n_{fi}^{eq}\rangle ={\left[H_{f},\frac{c_{pl}T_{f}u_{x}}{\phi },\frac{c_{pl}T_{f}u_{y}}{\phi },c_{sf}^{2}c_{pl}T_{f}+u_{x}^{2}c_{pl}T_{f},c_{sf}^{2}c_{pl}T_{f}+u_{y}^{2}c_{pl}T_{f}\right]}^{T}$$

In the system, ${\tilde{n}}_{f0}^{}$ is the only conserved quantity (${\tilde{n}}_{f0}^{}=n_{f0}^{}=H_{f}$). As the enthalpy $H_{f}$ is the basic evolution variable ($g_{fi}$ is now defined as the enthalpy distribution function), the shifting procedure $|{\tilde{n}}_{fi}^{}\rangle =N_{T}|n_{fi}^{}\rangle$ in the original thermal CLB equation should be modified. Explicitly, the modified shifting procedure is given by:

(28) $${\tilde{n}}_{f0}^{}=n_{f0}^{}\;{\tilde{n}}_{f1}^{}=-\frac{u_{x}c_{pl}T_{f}}{\phi }+n_{f1}^{}\;{\tilde{n}}_{f2}^{}=-\frac{u_{y}c_{pl}T_{f}}{\phi }+n_{f2}^{}\;{\tilde{n}}_{f3}^{}=u_{x}^{2}c_{pl}T_{f}-2\phi u_{x}n_{f1}^{}+n_{f3}^{}\;{\tilde{n}}_{f4}^{}=u_{y}^{2}c_{pl}T_{f}-2\phi u_{y}n_{f2}^{}+n_{f4}^{}$$

Formally, the collision process is still given by Equation (16), but it should be noted that it has been reconstructed since the shifting procedure is modified as Equation (28). The post-collision enthalpy distribution function $g_{fi}^{*}$ is determined via $|g_{fi}^{*}\rangle =M_{T}^{-1}|n_{fi}^{*}\rangle$, in which $|n_{fi}^{*}\rangle$ is given by:

(29) $$n_{f0}^{*}={\tilde{n}}_{f0}^{*}\;n_{f1}^{*}=\frac{u_{x}c_{pl}T_{f}}{\phi }+{\tilde{n}}_{f1}^{*}\;n_{f2}^{*}=\frac{u_{y}c_{pl}T_{f}}{\phi }+{\tilde{n}}_{f2}^{*}\;n_{f3}^{*}=u_{x}^{2}c_{pl}T_{f}+2\phi u_{x}{\tilde{n}}_{f1}^{*}+{\tilde{n}}_{f3}^{*}\;n_{f4}^{*}=u_{y}^{2}c_{pl}T_{f}+2\phi u_{y}{\tilde{n}}_{f2}^{*}+{\tilde{n}}_{f4}^{*}$$

Explicitly, $g_{fi}^{eq}$ in the velocity space is:

(30) $$g_{f0}^{eq}=H_{f}-\varpi_{1}c_{pl}T_{f}-\left(u_{x}^{2}+u_{y}^{2}\right)c_{pl}T_{f}\;g_{f1}^{eq}=\frac{\varpi_{1}}{4}c_{pl}T_{f}+\frac{1}{2}u_{x}c_{pl}T_{f}+\frac{1}{2}u_{x}^{2}c_{pl}T_{f}\;g_{f2}^{eq}=\frac{\varpi_{1}}{4}c_{pl}T_{f}+\frac{1}{2}u_{y}c_{pl}T_{f}+\frac{1}{2}u_{y}^{2}c_{pl}T_{f}\;g_{f3}^{eq}=\frac{\varpi_{1}}{4}c_{pl}T_{f}-\frac{1}{2}u_{x}c_{pl}T_{f}+\frac{1}{2}u_{x}^{2}c_{pl}T_{f}\;g_{f4}^{eq}=\frac{\varpi_{1}}{4}c_{pl}T_{f}-\frac{1}{2}u_{y}c_{pl}T_{f}+\frac{1}{2}u_{y}^{2}c_{pl}T_{f}$$

where $\varpi_{1}\in \left(0,\;1\right)$ and $c_{sf}^{}=c\sqrt{\varpi_{1}/2}=\sqrt{\varpi_{1}/2}$.

The enthalpy $H_{f}$ is defined by:

(31) $$H_{f}=\sum_{i=0}^{4}g_{fi}$$

Simultaneously, the temperature $T_{f}$ can be calculated by:

(32) $$T_{f}=\{\begin{array}{ll} H_{f}/\left(\sigma_{s}c_{pl}\right), & H_{f}\leq H_{fs}\\ T_{fs}+\frac{H_{f}-H_{fs}}{H_{fl}-H_{fs}}\left(T_{fl}-T_{fs}\right), & H_{fs}<H_{f}<H_{fl}\\ T_{fl}+\left(H_{f}-H_{fl}\right)/\left(\sigma_{l}c_{pl}\right), & H_{f}\geq H_{fl} \end{array}$$

where $T_{fs}$ and $T_{fl}$ ($T_{fs}\leq T_{fl}$) are solidus and liquidus temperatures, respectively; $H_{fs}$ and $H_{fl}$ are enthalpies corresponding to $T_{fs}$ and $T_{fl}$, respectively.

The liquid fraction $f_{l}$ is:

(33) $$f_{l}=\{\begin{array}{ll} 0, & H_{f}\leq H_{fs}\\ \frac{H_{f}-H_{fs}}{H_{fl}-H_{fs}}, & H_{fs}<H_{f}<H_{fl}\\ 1, & H_{f}\geq H_{fl} \end{array}$$

$\alpha_{ef}$ is defined as $\alpha_{ef}=k_{ef}/\left(\phi \rho_{l}c_{pl}\right)=c_{sf}^{2}\left(\zeta_{\alpha }^{-1}-0.5\right)\delta_{t}$.

### 3.2. Thermal MRT-LB Equation

Equation (4) can be rewritten as:

(34) $$\frac{\partial \left(c_{pm}T_{m}\right)}{\partial t}=\nabla \cdot \left(\frac{k_{em}}{\left(1-\phi \right)\rho_{m}}\nabla T_{m}\right)+\frac{h_{mf}a_{mf}\left(T_{f}-T_{m}\right)}{\left(1-\phi \right)\rho_{m}}$$

The MRT-LB equation for Equation (34) is:

(35) $$g_{mi}\left(x+e_{i}\delta_{t},\;t+\delta_{t}\right)-g_{mi}\left(x,\;t\right)=-{\left(M_{T}^{-1}QM_{T}\right)}_{ij}\left(g_{mj}-g_{mj}^{eq}\right)|{}_{\left(x,\;t\right)}+\delta_{t}S_{mi}^{}$$

where $g_{mi}\left(x,\;t\right)$ is the temperature distribution function of the metal foam, $g_{mi}^{eq}\left(x,\;t\right)$ is the equilibrium of $g_{mi}\left(x,\;t\right)$, and $Q=\mathrm{diag}\left(\eta_{T},\eta_{\alpha },\eta_{\alpha },\eta_{e},\eta_{e}\right)$ is the relaxation matrix.

The collision process of the evolution Equation (35) is implemented in raw-moment space as:

(36) $$|n_{mi}^{*}\rangle =|n_{mi}^{}\rangle -Q\left(|n_{mi}^{}\rangle -|n_{mi}^{eq}\rangle \right)+|{\tilde{S}}_{mi}^{}\rangle$$

where $|n_{mi}^{}\rangle =M_{T}|g_{mi}^{}\rangle$, $|n_{mi}^{eq}\rangle =M_{T}|g_{mi}^{eq}\rangle$, and $|{\tilde{S}}_{mi}^{}\rangle =M_{T}|S_{mi}^{}\rangle$.

The streaming process is executed in velocity space as

(37) $$g_{mi}\left(x+e_{i}\delta_{t},\;t+\delta_{t}\right)=g_{mi}^{*}\left(x,\;t\right)$$

where $|g_{mi}^{*}\rangle =M_{T}^{-1}|n_{mi}^{*}\rangle$. 

The equilibrium moment $|n_{mi}^{eq}\rangle$ is:

(38) $$|n_{mi}^{eq}\rangle ={\left[c_{pm}T_{m},0,0,\frac{\varpi c_{pm}T_{m}}{2},\frac{\varpi c_{pm}T_{m}}{2}\right]}^{T}$$

The equilibrium temperature distribution function $g_{mi}^{eq}\left(x,\;t\right)$ in the velocity space is given by:

(39) $$g_{mi}^{eq}=\{\begin{array}{ll} \left(1-\varpi_{2}\right)c_{pm}T_{m}, & i=0\\ \frac{1}{4}\varpi_{2}c_{pm}T_{m}, & i=1\sim 4 \end{array}$$

where $\varpi_{2}\in \left(0,\;1\right)$ is a tunable parameter of the D2Q5 lattice model.

Explicitly, the source term $|{\tilde{S}}_{mi}^{}\rangle$ is given by:

(40) $$|{\tilde{S}}_{mi}^{}\rangle ={\left[Sr_{m}+\frac{1}{2}\delta_{t}\partial_{t}Sr_{m},0,0,0,0\right]}^{T}$$

where $Sr_{m}=h_{mf}a_{mf}\left(T_{f}-T_{m}\right)/\left[\left(1-\phi \right)\rho_{m}\right]$.

The temperature $T_{m}$ is defined by:

(41) $$c_{pm}T_{m}=\sum_{i=0}^{4}g_{mi}$$

The effective thermal diffusivity $\alpha_{em}=k_{em}/\left[\left(1-\phi \right)\rho_{m}\right]=c_{sm}^{2}\left(\eta_{\alpha }^{-1}-0.5\right)\delta_{t}$, in which $c_{sm}=c\sqrt{\varpi_{2}/2}=\sqrt{\varpi_{2}/2}$ is the sound speed.

## 4. Numerical Simulations

**Figure 1:**
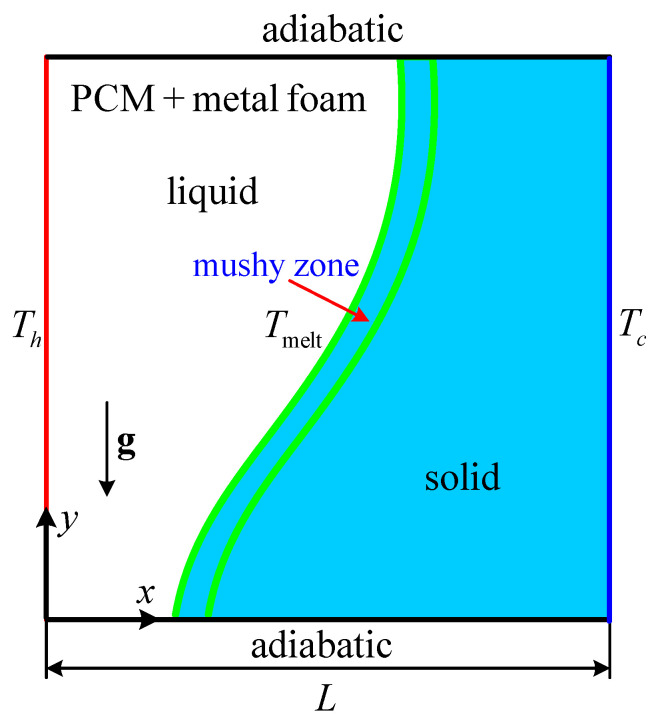
Schematic of melting with natural convection in metal foams.

In this section, the present enthalpy-based CLB model is employed to investigate solid-liquid phase change (melting) with natural convection in metal foams. The schematic of the problem under investigation is shown in Figure 1. Initially, the PCM (solid state) and metal foam are kept at equilibrium temperature $T_{i}$ ($T_{i}\leq T_{\mathrm{melt}}$). At t=0, the temperature of the left wall is raised to $T_{h}$ (*T_h_* > *T*_melt_), and consequently, the PCM starts to melt. The characteristic parameters are defined as follows:

(42) $$Da=\frac{K}{L^{2}},\;Ra=\frac{g\beta \Delta TL^{3}}{v_{fl}\alpha_{fl}},\;Pr=\frac{v_{fl}}{\alpha_{fl}},\;J=\frac{v_{e}}{v_{fl}},\;\hat{\sigma }=\frac{\rho_{m}c_{pm}}{\rho_{l}c_{pl}},\;\lambda =\frac{k_{m}}{k_{fl}}\;\Gamma =\frac{\alpha_{m}}{\alpha_{fl}},\;H_{v}=\frac{h_{mf}a_{mf}d_{p}^{2}}{k_{f}},\;Fo=\frac{t\alpha_{fl}}{L^{2}},\;St=\frac{c_{pl}\Delta T}{L_{a}}$$

where $\Delta T=T_{h}-T_{c}$ ($T_{h}>T_{c}$) is the characteristic temperature and $\alpha_{m}=k_{m}/\left(\rho_{m}c_{pm}\right)$.

In simulations, some required parameters are: $\delta_{x}=\delta_{y}=\delta_{t}=1$ (c=1), $c_{pl}=c_{ps}=1$, $\hat{\sigma }=1$, Pr=50, $H_{v}=5.9$, $d_{p}/L=0.0135$, $\lambda =\Gamma =10^{3}$, $k_{f}=0.0005$, J=1, and $\varpi_{1}=\varpi_{2}=1/2$. The effective thermal conductivities $k_{ef}$ and $k_{em}$ are simply determined by $k_{ef}=\phi k_{f}$ ($k_{f}=k_{fl}=k_{fs}$) and $k_{em}=\left(1-\phi \right)k_{m}$, respectively. To eliminate the unphysical numerical diffusion, the relaxation parameters $\zeta_{3}$ and $\zeta_{4}$ ($\zeta_{3}=\zeta_{4}=\zeta_{e}$) related to the second-order moments are determined by $\zeta_{e}=2-\zeta_{\alpha }$ [20,27]. The isothermal CLB equation [32] in conjunction with the volumetric LB scheme [36] is employed to solve the flow filed, and to realize the velocity and thermal boundary conditions, the non-equilibrium extrapolation scheme [37] is adopted. Each C++ serial program runs on a desktop computer (Inter(R) Core(TM) i5-8400 CPU@2.80GHz; RAM: 8.0 GB).

**Figure 2:**
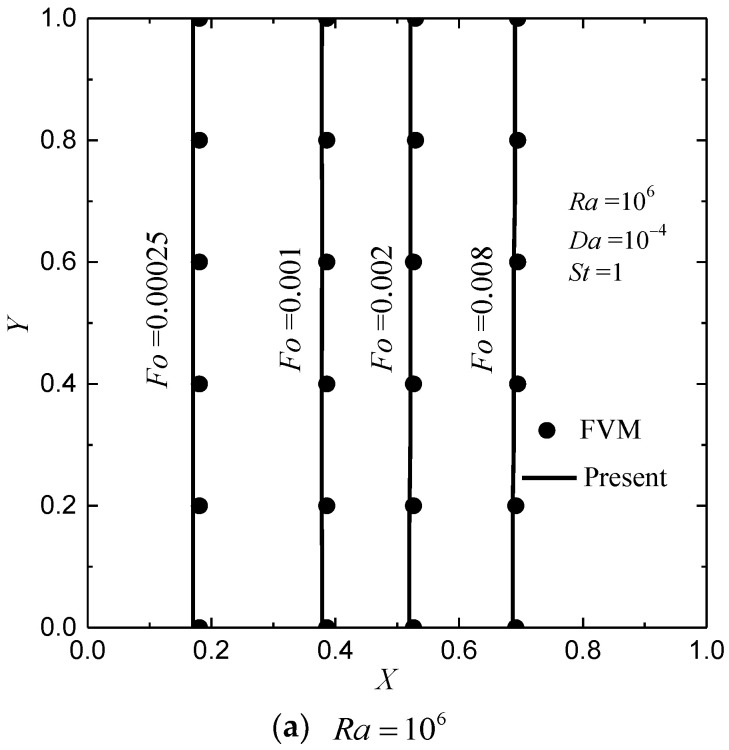

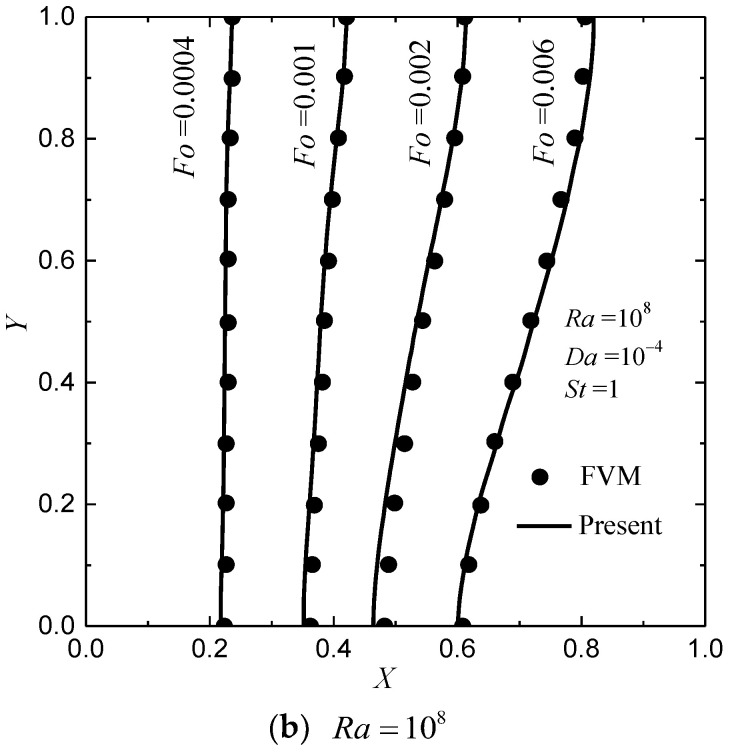
The melting front ($f_{l}=0.5$) at different values of Fo for $Ra=10^{6}$ and $10^{8}$ with $Da=10^{-4}$, ϕ=0.8 and St=1. (lines: present; symbols: FVM results [9]).

In Figure 2, the melting front at different values of Fo for $Ra=10^{6}$ and 10^8^ with $Da=10^{-4}$, ϕ=0.8 and St=1 are presented. For comparison purpose, the other parameters are chosen as $F_{\phi }=0.068$, $\theta_{h}=1$, $\theta_{i}=\theta_{c}=\theta_{0}=0$, and $\theta_{\mathrm{melt}}=0.3$ ($\theta =\left(T-T_{c}\right)/\Delta T$) [9]. For $Ra=10^{6}$, $N_{x}\times N_{y}=100\times 100$ is employed, and for $Ra=10^{8}$, $N_{x}\times N_{y}=300\times 300$ is employed. From the figure it can be found that the CLB results match well with the results obtained by FVM [9]. As show in Figure 2a, at $Ra=10^{6}$, the shape of the melting front is almost planar since the heat transfer inside the cavity is controlled by conduction due to the large value of metal foam-to-PCM thermal conductivity ratio. As Ra increases ($Ra=10^{8}$), the effect of natural convection becomes stronger, then the melting front moves faster near the top wall (see Figure 2b).

**Figure 3:**
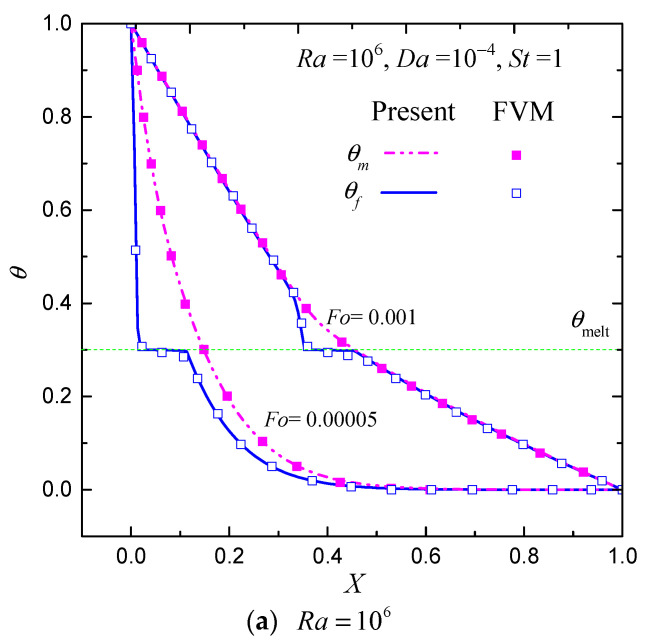

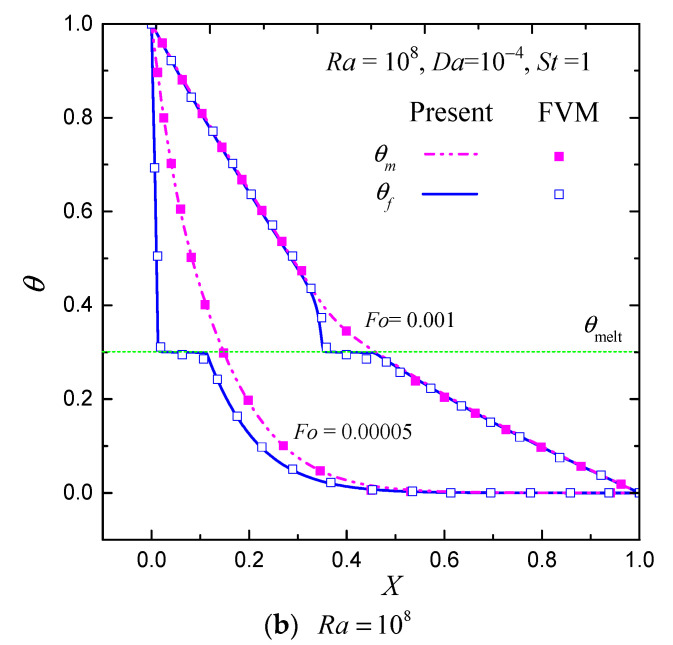
Temperature profiles at the mid-height of the cavity (Y=0.5) at different values of Fo for $Ra=10^{6}$ and $10^{8}$ with $Da=10^{-4}$, ϕ=0.8 and St=1. (lines: present; symbols: FVM results [9]).

In Figure 3, the temperature profiles (at Y=0.5) at different values of Fo for $Ra=10^{6}$ and $10^{8}$ with $Da=10^{-4}$, ϕ=0.8 and St=1 are shown. As can be seen from the figure, at the very beginning (Fo=0.00005), the thermal non-equilibrium effect is apparent (the temperature difference is very high). As Fo increases, the temperature difference progressively decreases due to the interfacial heat transfer. At Fo=0.001, the temperature difference tends to 0 except in the mushy zone, indicating that the thermal non-equilibrium effect at this stage is weak. As clearly illustrated in Figure 3, the temperature difference has a maximum value near the phase-change region (mushy zone). The FVM results [9] are also shown in Figure 3 for comparisons. It can be found that the CLB results are in good agreement with those results in Ref. [9]. 

**Figure 4:**
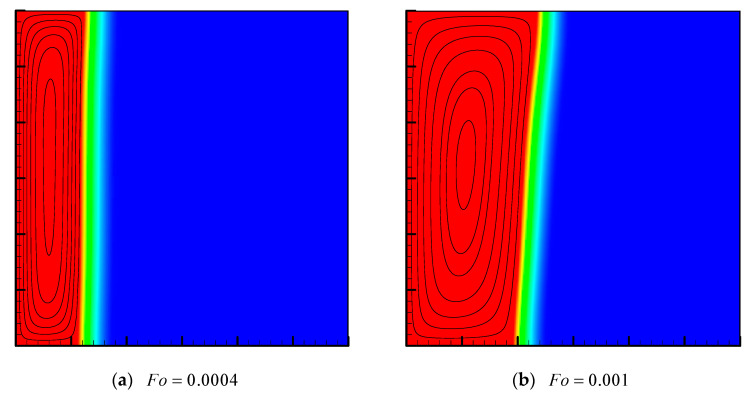

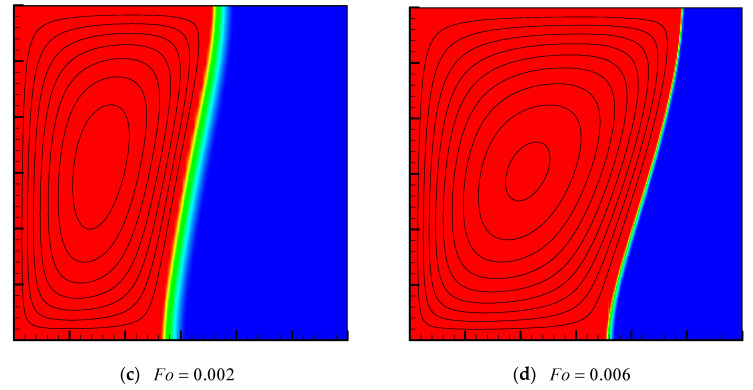
Streamlines with the phase field at different values of Fo for $Ra=10^{8}$ with $Da=10^{-4}$, ϕ=0.8 and St=1.

The flow field with the phase field at different values of Fo for $Ra=10^{8}$ with $Da=10^{-4}$, ϕ=0.8 and St=1 are presented in Figure 4. As shown in the figure, an elliptical shape vortex appears in the liquid region at Fo=0.0004. As Fo increases, natural convection effect becomes stronger, resulting in more hot liquid to move upwards, and consequently, the PCM melts faster in the upper region of the cavity. Although the Rayleigh number is high, the natural convection effect is weak since it has been severely suppressed by the effect caused by the metal foam’s ligament, and, during the melting process, the heat conduction through the high thermal conductive metal foam dominates the heat transfer. 

**Figure 5:**
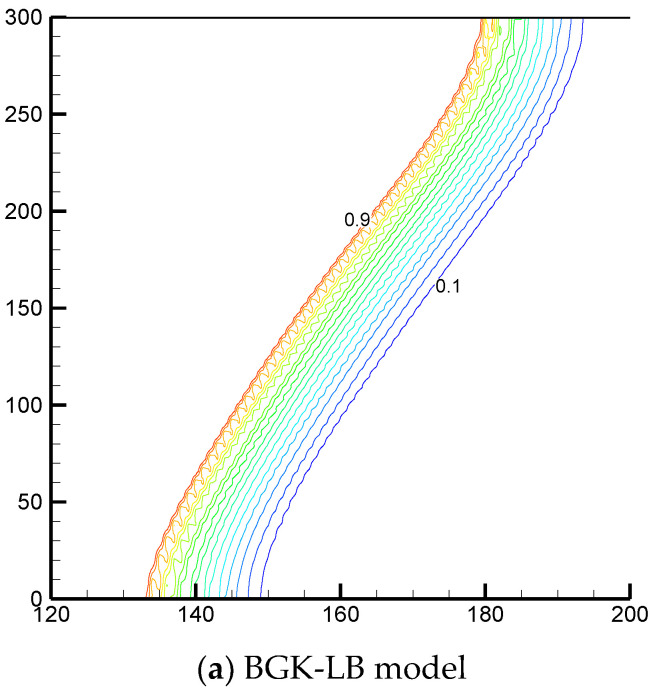

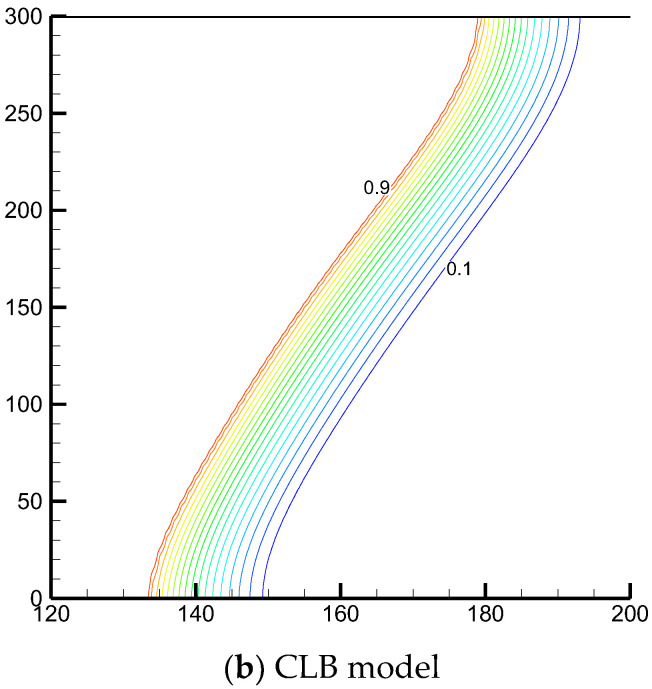
Local enlargement view of the liquid fraction distributions calculated by the BGK-LB and CLB models at Fo=0.002 for $Ra=10^{8}$ with $Da=10^{-4}$, ϕ=0.8 and St=1.

Since the cascaded collision model has tunable relaxation parameters as well as a high degree of Galilean invariance, it is expected that the unphysical numerical diffusion can be effectively eliminated in numerical simulations by using the CLB model. To confirm this statement, the liquid fraction distributions calculated by the CLB and BGK-LB models at Fo=0.002 for $Ra=10^{8}$ with $Da=10^{-4}$, ϕ=0.8 and St=1 are presented in Figure 5. The BGK-LB result means that the PCM’s temperature field is solved by the enthalpy-based BGK-LB equation (when $N_{T}$ is an identity matrix and $\zeta_{i}=1/\tau_{f_{l}}$, the enthalpy-based CLB equation degrades into the enthalpy-based BGK-LB equation with $\alpha_{ef}=c_{sf}^{2}\left(\tau_{f_{l}}-0.5\right)\delta_{t}$). As can be seen in Figure 5a, the liquid fraction distribution calculated by the BGK-LB model exhibits significant numerical oscillations because the BGK-LB model has no free relaxation parameters to eliminate the effect of numerical diffusion on numerical simulations. On the contrary, the effect of numerical diffusion is almost invisible in Figure 5b because the CLB model has free relaxation parameters to eliminate such an effect. By setting $\zeta_{e}=2-\zeta_{\alpha }$, the effect of the numerical diffusion on numerical simulations can be effectively eliminated by the present CLB model.

**Figure 6:**
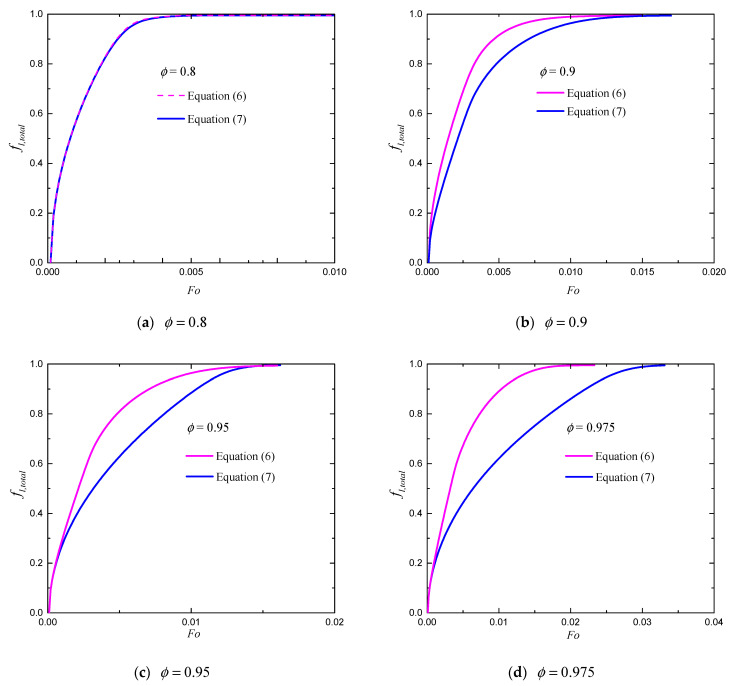
The total liquid fractions for different porosities with $Ra=10^{8}$ and St=1.

$F_{\phi }$ and K are functions of the structure of the porous media. Equation (6) is proposed for packed beds of spherical particles based on Ergun’s experimental study, while Equation (7) is proposed for high porosity metal foams such as aluminum foam [33,34]. In what follows, the empirical correlations for $F_{\phi }$ and K given by Equations (6) and (7) are evaluated. In numerical simulations, the related parameters are set as $\theta_{h}=1$, $\theta_{i}=\theta_{c}=\theta_{\mathrm{melt}}=\theta_{0}=0$. Numerical simulations are conducted based on $N_{x}\times N_{y}=100\times 100$. The total liquid fractions $f_{l,total}$ for different porosities with $Ra=10^{8}$ and St=1 are presented in Figure 6. For a low value of porosity (ϕ=0.8), the total liquid fraction $f_{l,total}$ predicted with Equation (6) is almost the same as that predicted with Equation (7). As the porosity increases, the two empirical correlations yield different results (see Figure 6b–d). When $F_{\phi }$ and K are given by Equation (6), the melting rate of the PCM is faster than that predicted with Equation (7). This can be expected because the permeability given by Equation (6) is much larger than that given by Equation (7) at high porosities, leading to a stronger convection effect which promotes the heat transfer during melting process. Hence, for high porosity metal foams, appropriate empirical correlations should be employed. Since Equation (6) is proposed for packed beds, it overestimates the melting rate of the PCM. The empirical correlations given by Equation (7) are expected to provide a good estimate of the practical situations. 

**Figure 7:**
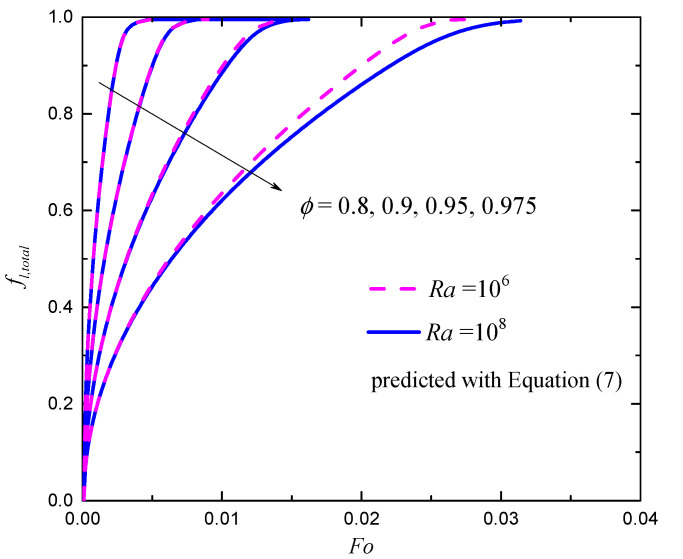
The total liquid fractions for different porosities with $F_{\phi }$ and K given by Equation (7).

In Figure 7, the total liquid fractions of $Ra=10^{6}$ and $10^{8}$ for different porosities with $F_{\phi }$ and K given by Equation (7) are shown. As can be seen in the figure, when ϕ increases, the PCM’s melting rate decreases. An increase in the metal foam’s porosity leads to stronger convection effect of the liquid; however, it also reduces the effect of conduction through the metal foam’s ligament. This observation denotes that the total heat transfer from hot (left) wall is dominated by heat conduction through the high thermal conductive metal foam. The comparison between the total liquid fractions of $Ra=10^{6}$ and $10^{8}$ indicates that the effect of Ra on melting is weak because the convection has been severely suppressed by the metal foam’s ligament. Although smaller porosity increases the PCM’s melting rate, it also denotes smaller volume of PCM for LHS. A further optimization investigation on porosity of the metal foam to balance the PCM’s melting rate and LHS capacity is quite necessary.

## 5. Conclusions

An enthalpy-based CLB model is constructed for investigating heat transfer in solid-liquid PCMs with metal foams. The effects of the inertial coefficient, permeability and porosity on phase-change processes are examined. The findings of this research are:

(1) The melting front and temperature profiles at different Fourier numbers predicted by the CLB model match well with the available data in previous studies, demonstrating the effectiveness and practicability of the CLB model for investigating heat transfer in solid-liquid PCMs with metal foams.
(2) The empirical correlations of $F_{\phi }$ and K given by Equation (6) based on packed beds overestimate the PCM’s melting rate when the metal foam’s porosity is high, while the empirical correlations for metal foams such as aluminum foam given by Equation (7) are expected to provide a good estimate of the practical situations.
(3) The PCM’s melting rate increases as the metal foam’s porosity decreases since the total heat transfer from the hot wall is dominated by heat conduction through high thermal conductive metal foam. Moreover, the effect of the Rayleigh number on phase-change process is weak since it has been severely suppressed by the metal foam’s ligament.
(4) Although smaller porosity increases the PCM’s melting rate, it also reduces the volume of PCM for LHS, leading to a lower LHS capability. A further optimization investigation on metal foam’s porosity to balance PCM’s melting rate and LHS capacity is quite necessary.

## Nomenclature

$\hat{\sigma }$	heat capacity ratio (metal foam-to-PCM)
c	lattice speed, $c=\delta_{x}/\delta_{t}=1$
$c_{sf}^{}$	sound speed, $c_{sf}^{}=c\sqrt{\varpi_{1}/2}=\sqrt{\varpi_{1}/2}$
$c_{sm}$	sound speed, $c_{sm}=c\sqrt{\varpi_{2}/2}=\sqrt{\varpi_{2}/2}$
T	temperature
g	gravitational acceleration
$d_{p}^{}$	mean particle diameter or mean diameter of the pores
p	pressure
$d_{f}^{}$	fiber diameter of metal foam
Da	Darcy number
$e_{i}$	discrete lattice velocity
F	total body force
L	characteristic length
$f_{l,total}$	total liquid fraction
Fo	Fourier number
**G**	buoyancy force
$k_{em}$	effective thermal conductivity (metal foam)
$H_{v}$	volumetric heat transfer coefficient
J	viscosity ratio
$M_{T}$	transformation matrix
$N_{T}$	shift matrix
Ra	Rayleigh number
St	Stefan number
$T_{0}$	reference temperature
$T_{h}$	hot wall’ temperature
λ	thermal conductivity ratio (metal foam-to-PCM)
$T_{c}$	cold wall’s temperature
$T_{i}$	initial temperature
$T_{\mathrm{melt}}$	melting temperature
$k_{f}$	thermal conductivity (PCM)
u	velocity
X,Y	coordinates, X=x/L, Y=y/L
$c_{p}$	specific heat
$k_{d}$	thermal dispersion conductivity
$a_{mf}$	specific surface area
$k_{ef}$	effective thermal conductivity (PCM)
$h_{mf}$	interfacial heat transfer coefficient
$f_{l}$	liquid fraction
$L_{a}$	latent heat
$F_{\phi }$	inertial coefficient
K	permeability
Pr	Prandtl number
$\alpha_{fl}$	Thermal diffusivity (liquid PCM)
$\alpha_{ef}$	effective thermal diffusivity (fluid)
$\alpha_{em}$	effective thermal diffusivity (metal foam)
β	thermal expansion coefficient
ρ	density
ϕ	porosity of metal foam
*v_e_*	effective kinematic viscosity
$v_{fl}$	kinematic viscosity (liquid PCM)
$\varpi_{1}$, $\varpi_{2}$	model parameters
*k_m_*	thermal conductivity (metal foam)
$\delta_{x}$	lattice spacing
$\delta_{t}$	time step
Γ	thermal diffusivity ratio (metal foam-to-PCM)
Subscripts	
f	fluid (PCM)
m	metal foam
l	liquid PCM
s	solid PCM
